# Supporting self-recovery in post-conflict situations: a case study of Syria

**DOI:** 10.1186/s41018-022-00125-y

**Published:** 2022-08-03

**Authors:** Taylor Raeburn-Gibson

**Affiliations:** Universitat Internacional de Catalunya School of Architecture, Edifici Alfa - Campus Barcelona, Immaculada, 22, 08017 Barcelona, Spain

**Keywords:** Self-recovery, Post-conflict, Housing, Reconstruction, Shelter, Humanitarian aid

## Abstract

Supporting shelter self-recovery is a modality of humanitarian aid which remains ill-defined and misunderstood despite the many aid organizations that utilize this approach. Of the little existing knowledge and best practices regarding self-recovery support methods, most has been developed for natural disaster contexts, and not post-conflict. Post-conflict situations are much more complex than disasters due to a multitude of economic, social, and other factors. Further research is greatly needed to support self-recovery programs in post-conflict situations, especially due to the complexities involved. This research aims to highlight the unique complexities of post-conflict support to self-recovery and to identify ways of improving this support. This is done through a combination of a literature review and a case study of self-recovery support methods currently being employed in Syria. The results include a framework which identifies and categorizes common factors, barriers, and facilitators which influence the implementation of self-recovery support projects. The results also include a list of recommendations to improve these projects for stakeholders involved. Based on an analysis of these recommendations, five Key Areas for Action are discussed which are as follows: maximizing implementing organizations’ capacities, contextualizing risks, increasing adaptable and flexible programming, addressing the social dimension, and improving international coordination.

## Introduction

The role of housing within humanitarian action is being acknowledged to have increased importance related to social and economic outcomes such as health, livelihoods, and social cohesion (Interaction 2020). One specific shelter programming approach that has shown to produce improved social and economic outcomes is *self-recovery support* (Barakat 2003; Maynard et al. 2017; Maynard and Parker 2018). Self-recovery support is a newer term, but is not a new concept, as similar approaches have previously been described as *self-help*, *self-build*, and *owner-driven reconstruction* (Turner 1976; Davis 1978; Hamdi 1995; Harris 1998; Barakat 2003). Currently, organizations are leading self-recovery support programming despite the paucity of research and guidance on best practices (Twigg et al. 2017). This is especially true for post-conflict^1^ situations (Flinn et al. 2017; Parrack et al. 2017; Schofield and Flinn 2018). Organizations require support in understanding the uniqueness and implications of leading self-recovery support programming in post-conflict situations.

Self-recovery support involves aid or development organizations providing assistance to enable households to rebuild or repair their homes themselves or using local skills and techniques (Parrack et al. 2014; Maynard et al. 2017; Newby 2018; Schofield and Flinn 2018). This may include financial, technical, and material assistance. For implementing organizations, leading self-recovery programming is difficult and complex. To support self-recovery, organizations must consider factors such as vernacular architecture and local building regulations, alternate financing systems, decentralized monitoring and controlling mechanisms, participative program design, and consultation with professionals such as engineers and architects (Corsellis and Vitale 2005; Maynard et al. 2017). This is notably more complex for an implementing organization than traditional responses such as planned camps. Self-recovery support also demands buy-in and engagement from a diverse group of stakeholders including local authorities, aid-coordinating bodies, the private sector, donors, community leaders and groups, and, most importantly, the homeowners themselves. The complexities involved in this type of support necessitate a strong and capable organization with robust manpower, funding, technical knowledge, and permanence (Davis 2015).

Self-recovery support is important to understand due to its implications in terms of coverage of response and funding. Barakat (2003) showed that in post-conflict responses in the Balkans, self-help approaches were demonstrated to cost 40% less than contractor-led projects (Barakat 2003, pp. 33-35). With lower cost programming, self-recovery support approaches allow funds to reach more affected people. This is important at a time where most post-disaster housing recovery responses only reach up to 20% of those affected (only 10.4% in the 2010 Haiti earthquake) (Parrack et al. 2014). In conjunction, the humanitarian sector has become increasingly under-funded. The funding gap in the sector is now over four times what it was just one decade ago, and is the highest ever, reaching 46% in 2021 (Skretteberg 2019; OCHA 2022b).

Leading self-recovery support programming in post-conflict contexts comes with additional complexities when compared to post-disaster situations. Whereas in disaster contexts the threat has come and gone, and the population is then generally united in its recovery, post-conflict situations are more ambiguous. Post-conflict situations include added complexities of underlying social conflicts, much blurrier transitions between emergency and recovery, the frequent loss of tenure documentation, and the risk of continued hostilities suppressing international support (Barakat 2003; Corsellis and Vitale 2005; Davis 2015; Humanitarian Coalition 2015). The challenges that implementing organizations face when implementing this support in post-conflict situations can be classified in levels: (1) economic, (2) social, (3) governance, (4) legal, (5) contextual, and (6) technical. At each of these levels, there exist barriers that can impede the use of this approach and facilitators that can facilitate this approach.

The implementation of self-recovery support in post-conflict situations must be dictated by the humanitarian principles. Humanitarian stakeholders leading this modality of support must work closely with local stakeholders to ensure that targeted beneficiaries are selected without political, economic, or other biases. This requires delicate negotiation with local authorities, a stakeholder whose support is key in implementing self-recovery support. Self-recovery support modalities must also uphold the principle of humanity, ensuring vulnerable households are prioritized. Since active participation of households is cornerstone of self-recovery programming, the ability for vulnerable households to actively participate in shelter works must be considered and accounted for. Selection criteria for inclusion in these programs must also be clearly set and communicated so that beneficiaries are selected based on need and without discrimination.

Self-recovery support remains under-researched and unsupported by any comprehensive guidelines (Twigg et al. 2017). This is despite the use of this approach by numerous organizations both in disasters and conflicts (IOM, UNHCR and Shelter Centre 2018; Maynard and Parker 2018; Newby 2018; Schofield et al. 2019). This includes a lack of case studies and data from the field as best practices “[remain] poorly understood” (Schofield and Flinn 2018, p. 29). The current research that exists has been developed strictly for the disaster context (Flinn et al. 2017; Schofield and Flinn 2018). In one recent study, Opdyke et al. (2020) note that humanitarian shelter and settlements experts identified self-recovery as being one of the top research priorities moving forward for the sector. Additionally, the Global Shelter Cluster has recently included both self-recovery and conflict within their priority research areas (Parrack 2020). This research aims to highlight the unique considerations for implementing post-conflict self-recovery support and to provide recommendations to implementing organizations to improve their programming. To accomplish this, the research identified barriers and facilitators which affect the implementation of self-recovery support programs. This was done through a review of relevant literature combined with primary qualitative data collection from implementing organizations working in Syria. The literature review allowed to identify the major factors involved in supporting self-recovery and to group them in levels, thus forming a framework. The case study then added a further layer of knowledge to the framework through the confirmation of factors previously identified and the inclusion of new factors. Based on the understanding of influencing factors from a holistic approach, recommendations and Key Areas for Action were identified to improve the self-recovery process for specific stakeholders involved.

### Country context

The Syrian civil war has created the most significant humanitarian crisis in the past decade. There are over two million internally displaced people (IDPs) living in informal settlements and planned camps in Syria (OCHA 2022a). Shelter is the most pressing need currently in Syria among IDPs with 20% of housing in the country being damaged as of 2017 (OCHA 2020, p. 2; World Bank Group 2017, p. 21). The demand for shelter in Syria is expected to rise in the future with more Syrians planning on returning in the near future. In 2022 alone, 250,000 IDPs are expected to return to Syria (OCHA 2022a). As noted by one participant in this study, COVID-19 has also propelled Syrians to return home over fears of the spread of the virus in IDP camps (A. Dehny, skype interview, April 16, 2020)). As more returnees arrive to Syria, the demand for shelter will continue to increase.

Shelter response activities in Syria are currently coordinated by two main Shelter Cluster hubs: the Turkey hub in Gaziantep and the Syria hub in Damascus. From Gaziantep, UNHCR coordinates 143 member organizations in a cross-border aid operation targeted at IDPs within the opposition-controlled areas of Syria (Global Shelter Cluster 2022). Some shelter support is also coordinated through UNHCR in Damascus and from various organizations in Jordan.

There are many factors complicating the shelter response in Syria which are related to the conflict. Governance is one key persisting challenge to humanitarian actors. The Syrian government has restricted the access of aid organizations, suffered from corruption and a lack of transparency, used land legislation to erase opposition communities and to enhance pro-regime ones, and co-opted humanitarian funding to advance its own interests (Yazigi 2017; Sparrow 2018; Dacrema and Talbot 2019; Kayyali 2019). Funding is also a key concern for aid organizations in Syria as in 2021, Syria remained 56% underfunded for inter-agency appeals (OCHA 2022b). One final key challenge for shelter operations is the difficulty in confirming tenure documentation for homeowners due to land registries being damaged or destroyed and families losing their property documentation during the conflict (NRC 2016).

There are a variety of aid organization-led shelter response modalities currently being employed in Syria. These include shelter repairs and rehabilitations, collective shelter upgrades, the distribution of non-food items (NFIs) and shelter kits, and emergency shelter provision (Global Shelter Cluster 2022). As no detailed definition exists for self-recovery support activities, three levels of classification were created. These levels (see Table 1) help to understand which of the above range of shelter responses currently being used in Syria can be considered as supporting self-recovery. The levels represent how much agency the beneficiaries have over the reconstruction process, with the higher levels providing the most agency to homeowners.

**Table 1:** Classification of levels of supporting self-recovery of current shelter response modalities being employed in Syria

Supporting self-recovery level	Shelter response modality
Level I	Provision of shelter kits: This method involves the distribution of tools and materials kits by aid organizations. Although in many cases shelter kits are only used for tents and emergency shelter, in some cases, homeowners use these kits to repair their homes, which can thus be considered self-recovery support.
Level II	Contractor-led shelter rehabilitations: This method involves an aid organization hiring a contractor to conduct the repairs for the homeowner. This is only considered to be self-recovery support if the homeowner has decision-making power and is involved in the process.
	Cash-for-work rehabilitations: This method involves the aid organization managing the project themselves and paying workers directly. This was not found to be used frequently.
Level III	Homeowner-led shelter rehabilitations: This method involves homeowners being given cash directly from aid organizations for the repairs. Homeowners then either complete the work themselves or hire their own local contractors or labor.

All of these shelter response modalities can be considered as supporting self-recovery because they enable households to repair their homes by themselves or through local labor, and these modalities each provide some level of agency to the homeowner (Maynard et al. 2017; Newby 2018). Self-recovery support’s traditional three-prong approach of material, financial, and technical support is applicable within each of these three levels. This classification system is only applicable in the context of Syria. Levels I responses are not discussed significantly in this research but were included to show the range of responses that can be considered as supporting self-recovery.

This classification helped to identify that the higher levels of self-recovery support in Syria consisted of shelter rehabilitation modalities. Shelter rehabilitation programs are a commonly used modality of support currently in Syria. This modality is supported by the Shelter Cluster’s Shelter Repair and Rehabilitation Guidelines, published by IOM, UNHCR, and Shelter Centre, which are some of the only guidelines currently of this kind. Of the 56 organizations that submitted appeals for funding as per the 2019 Syria Humanitarian Response Plan (HRP), 45 organizations listed shelter rehabilitation as a response, which equates to 80% of all organizations. The demand for shelter rehabilitation support in Syria is high, with 157,000 people in need of this modality of support in Northwest Syria alone as of May 2020 (OCHA 2020, p. 2).

## Methods

A combination of secondary and primary research methods were implemented. Secondary research was conducted through a literature review of resources pertaining to self-recovery within post-conflict contexts. This enabled the identification of barriers and facilitators relating to the implementation of self-recovery support projects. These were then analyzed and categorized into factors, and the factors then into six levels: economic, social, governance, legal, contextual, and technical. These barriers, facilitators, factors, and levels were put into a table to form the Literature Framework.

Primary research was also conducted to form a case study of Syria. This included semi-structured qualitative interviews and electronic questionnaires administered from March to April 2020 with participants from aid organizations working in shelter response in Syria. Interviews were conducted online over video calls (in English) with each interview lasting between 20 and 80 minutes. Open-ended interview questions were used to understand common barriers and facilitators to self-recovery projects, lessons learned, and recommendations on improving self-recovery programming. A total of 14 semi-structured interviews and 12 questionnaires were completed throughout March and April 2020. The data collected was then transcribed, cleaned, and analyzed to confirm and identify further factors, barriers, and facilitators which were added to six levels of the Literature Framework. Combined, this formed a Post-Conflict Supporting Self-Recovery (PCSSR) Framework which included data from both the literature and case study. Recommendations for improving self-recovery support projects were then determined based on analysis of the PCSSR Framework. A Recommendations table was then created and grouped into the same six levels as in the frameworks. The recommendations sought to highlight best practices (activating/propelling facilitators), propose solutions to barriers, and to consider some direct recommendations from some participants. Lastly, the Recommendations table was analyzed to identify key cross-cutting areas for action which emerged from the recommendations.

Participants were selected for this study with the goal of including a variety of actors involved in supporting self-recovery in the case study location, Syria. The interviews were conducted remotely due to COVID-19 travel restrictions and security considerations which limited the ability to include certain stakeholder such as government officials. Consequently, the research focused on maximizing the representation of NGO and IGO participants. NGOs and IGOs were selected from the list of organizations conducting shelter responses in Syria according to the 2019 UN OCHA Syria HRP appeals for funding. Participants within these organizations were selected only if they had comprehensive knowledge of their organization’s shelter programs in the field. Participants included shelter specialists, operations managers, project managers, and program coordinators and managers. A snowball method was used during the interviews which led to further contacts with organizations specifically conducting self-recovery work.

Other participants included a private engineering firm representative and a large donor. The engineering firm was sought out to discuss the potential for private sector involvement in self-recovery projects. The donor was engaged to help understand the identified barriers and facilitators from their perspective. Because of the small number of private sector and donor participants, information from these participants was used only to confirm and validate information given by the NGOs and IGOs and no specific barriers or facilitators were drawn directly from this data. Table 2 shows the full list of participants.

**Table 2:** Research participants

#	Data collection format	Type of organization	Name of organization	Name of participant
1	Interview	IGO	IOM	-
2		IGO	-	Henri Stalder
3		IGO	Violet Organization / UNHCR Turkey Hub Strategic Advisory Group	Asmahan Dehny
4		IGO	UNHCR - Damascus Hub	-
5		INGO	Caritas Luxembourg	-
6		INGO	Norwegian Refugee Council	Gareth Lewis
7		INGO	Qatar Charity	Amro Katkhada
8		INGO	Qatar Red Crescent Society	-
9		INGO	World Vision International	-
10		INGO	-	Joud Keyyali
11		NNGO	Violet Organization	Omar Shami
12		NNGO	Social Development International	Muhammad Yasin
13		NNGO	SARD	Fares Al Saleh
14		Private	Arup Group	-
15	Questionnaire	IGO	UN-Habitat	-
16		INGO	Medair	-
17		INGO	Cordaid	-
18		INGO	Agency for Technical Cooperation and Development	-
19		NNGO	Syria Relief	-
20		NNGO	Syrian Engineers for Construction and Development	-
21		NNGO	Syria Relief and Development	-
22		NNGO	Ahl Horan Organization	-
23		INGO	Mercy Corps	-
24		INGO	Danish Refugee Council - Damascus Office	-
25		Donor	Office of Foreign Disaster Assistance	-
26		INGO	ONG Rescate International	-

A dash (-) means that the organization or participant indicated they did not want their name to be shared for confidentiality reasons

Twenty-four different organizations participated in this study. Organizations were selected to ensure a distribution of national NGOs (NNGOs), international NGOs (INGOs), and intergovernmental organizations (IGOs). Organizations were also selected to ensure a distribution of those working in government-controlled and opposition-controlled areas of Syria. This is an important distinction due to the differences in oversight and regulation of shelter programming in these different areas.

## Results

**Fig. 1:**
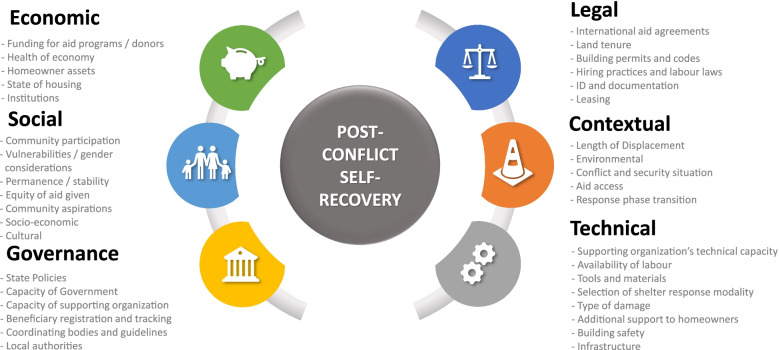
Simplified PCSSR Framework: factors affecting post-conflict self-recovery support across the six levels. Bold black text = levels. Gray text = factors

The final PCSSR Framework was formed with 36 pieces of literature and the case study data. This framework included 244 identified barriers and facilitators, grouped into 37 factors across six levels: (1) economic, (2) social, (3) governance, (4) legal, (5) contextual, and (6) technical. Figure 1 offers a simplified version of the PCSSR framework, showing simply the 37 factors across the six levels.

Below is a summary of the key barriers, facilitators, and factors identified in the PCSSR Framework.

At the first level, various economic factors were noted to affect post-conflict self-recovery support programming. The key factor at the economic level is funding for aid programming. Key barriers noted from the Syria study included donor concerns over conflict risk, trust of NNGOs, and the risk of interventions causing permanent demographic changes to the communities. Donors were also noted to show a general mistrust of cash programming in the post-conflict environment due to the perception that homeowners will not spend the cash on shelter due to other priorities. Additional barriers related to donors include the fact that donors do not often tend to support self-recovery modalities, preferring instead projects where they can reach more beneficiaries. Finally, donors are hesitant to support longer-term shelter interventions due to the risk of the conflict re-escalating. One facilitator identified was that donors are, in general, becoming more supporting of cash programming.

Another factor identified at the economic level was the health of the economy. Fluctuating material prices and currency inflation, common in many conflict situations, were noted to cause issues specifically for self-recovery projects which can have longer timeframes. Housing stock was also identified as a barrier due to the lack of maintenance of housing in prolonged displacements. Finally, the state of economic institutions can hamper self-recovery programming as banks can often be crippled due to the conflict, this making cash transfers difficult and costly.

At the social level, community participation was identified as a key factor. Conflicts can lead to the disappearance of social organizations and networks, lingering social tensions from the conflict, and homeowners not wanting to remain in their homes due to psychological trauma. Despite these challenges, participation of the community in self-recovery programs can have great benefits to the post-conflict recovery of societies. Facilitators identified to enable these approaches included engaging the community through focus group discussions, working with community leaders and institutions, and providing training to homeowners. One specific method employed was to provide community-based milestones in order for homeowners to receive subsequent payments, thus encouraging homeowners to support each other the community.

Gender considerations are also a key factor as conflicts often result in a lack of men due to the men being involved in the conflict. This can result in women taking on further responsibilities and can lead to some women and children being more vulnerable and sometimes not able to lead household repairs themselves. One facilitator identified related to gender considerations was to prioritize women who are vulnerable through proper beneficiary selection criteria. Another facilitator was to employ young men so that they can actively engage in the construction to help them reintegrate back into the community when they return from fighting.

At the governance level, barriers were noted relating to state policies and the capacity of governments. State policies can be very prohibitive in post-conflict situations such as policies that prohibit international aid, policies that prohibit the return of homeowners, and laws prohibiting or mandating certain types of reconstruction. Government capacities can also be greatly reduced due to the conflict resulting in a lack of clear government planning, policies, and institutions to support construction works. In addition, corruption and private sector lobbying can complicate reconstruction efforts. Finally, government biases towards certain ethnic groups can affect approvals for reconstruction. At the local authority level, other barriers include issues with approvals and pressure from the authorities onto how organizations conduct their programming. To mitigate this, negotiations with the government to streamline approvals processes and memorandums of understanding with local or state governments can be beneficial.

The capacity of the implementing organization is a particularly important factor identified at the governance level. The case study showed that difficulties with coordinating and monitoring the work were a key barrier for organizations. This was largely due to the security situation and staffing constraints. Complicating monitoring further was limited Internet connectivity in the field. Competing priorities within the organization also affected self-recovery being selected as a response modality, as more immediate emergency response such as distributing NFIs often took priority. Finally, it was noted that some previous organizational knowledge was acting as a barrier since it was not being properly contextualized to the location. This included the *Build Back Better* concept which means to build back a stronger and safer shelter than was there previously. Although this concept is applicable in post-disaster responses, in post-conflict there may be no need for such an approach. That said, previous organizational experience in leading self-recovery programming, either from post-disaster or post-conflict contexts, was shown as a clear benefit to implementing this support in post-conflict situations. Another key facilitator to implementing organizations was the use of contractors or implementing partners to oversee constructions. This greatly reduces the burden on the managing organization as it improves the ease of coordination and monitoring.

At the legal level, the key factor identified was land tenure. One barrier to implementing self-recovery support was the destruction of land registries and loss of land tenure documentation due to the conflict. Another barrier was the general complexity of the housing, land, and property (HLP) system in post-conflict states. A key facilitator identified to ease tenure verification in post-conflict situations is for implementing organizations to accept alternative tenure documentation such as utility bills or statements from community leaders. One other factor identified at the legal level was building permits. Due to conflicts resulting in crippled institutions which have limited capacity to manage systems such as building permitting, approvals for self-recovery projects can be greatly delayed.

Key factors at the contextual level were identified to be the security situation and access of aid organizations. The security situation is one of the most difficult challenges as conflicts can quickly escalate and shift geographically. Other barriers relating to security include a lack of security forces to protect aid agencies and policies of aid agencies being too restrictive in terms of initiating projects in dangerous areas. Another barrier, especially for donors, is the risk that repaired housing can be re-damaged during a subsequent conflict or even taken over control of by other parties. These security challenges can lead to some aid agencies prohibiting any presence in areas with security risks. As in Syria, this can result in the need for cross-border responses. Cross-border responses greatly increase the complexity of managing self-recovery support since this modality requires continuous monitoring and controlling. One facilitator identified in the case of cross-border support is the presence of local organizations in-country which can be partnered with to implement the support on behalf of the international aid organization. Local professionals in these organizations possess superior technical knowledge of the housing construction and are able to effectively oversee constructions.

The length of displacement of the population is also a key contextual factor. The longer populations have been displaced, the more of a challenge this becomes to support self-recovery. This is due to the potential for loss of HLP documentation, the secondary occupation of homes while displaced, and the overall lack of upkeep of the property over the length of displacement.

Finally, at the technical level, a key factor identified was the type of housing damage caused by the conflict. This is a key barrier since the complex types of damage caused by military ordnance are not well-understood. The lack of guidelines, standards, and manuals to support the repair of this type of damage are lacking from aid organizations, governments, and the private sector. The type of damage caused by conflicts can often be structural in nature, such as damage to columns, slabs, or foundations. The presence of structural damage was a key barrier to self-recovery programs as this damage cannot be repaired without strict engineering oversight. Additionally, this type of repair comes with the perception of being reconstruction, which is not considered the place of aid organizations and that of the government. To facilitate this, the case study showed that damage classification scales can be used to effectively identify houses that can and cannot be included in self-recovery support programming. Finally, the case study also showed that conflicts can result in other secondary damages such as those due to people looting houses for copper wire or other materials.

The implementing organization’s technical capacity is a key factor at the technical level. This can be a barrier when there is a lack of professionals such as engineers on staff to oversee constructions. Due to the damage type during conflicts, engineers are key to evaluating damage and supporting repairs. Engineers must have access to the homes to monitor them throughout constructions.

Other factors at the technical level include building safety, tools and materials available, and other infrastructure considerations. Building safety was identified as a major barrier due to the uncertainty of structural components of buildings following conflict damage. Also, the potential presence of unexploded ordnance (UXOs) is a barrier as aid organizations are not equipped to handle these. Tools and materials available are another key consideration as local markets can be severely impacted by the conflict and purchasing from international markets comes with long delays, cost increases, and complications due to uncertain importing regulations. A facilitator to address this was the pre-positioning and provision of select tools and materials for homeowners which they cannot access through local markets. The lack of other infrastructure systems such as water, electricity, sewage, and transportation was also noted as a key barrier. This creates challenges to the aid organization since these other repairs will require additional funds or coordination with other stakeholders. Multisectoral programming approaches, or area-based approaches, were one method identified that can address this challenge.

Following the PCSSR Framework, the Recommendations table (see Table 3) includes 57 recommendations directed towards various stakeholders involved in self-recovery support. These recommendations were developed to counter the barriers and promote the facilitators identified in the PCSSR Framework. Thus, these recommendations were formed from analysis of both literature and the Syrian case study. The stakeholders considered for these recommendations include NNGOs, INGOs, IGOs, donors, the IASC Global Shelter Cluster, universities, and engineering firms. These recommendations provide stakeholders specific guidance in their implementation of self-recovery support in post-conflict situations. Each recommendation is given an ID code (#x#) which will be used in the discussion to reference specific recommendations (recs). The recommendations were grouped into Key Areas for Action and will be further discussed within the discussion section.

**Table 3:** Recommendations table

Level	Recommendation topic	Recommendation ID code	Conclusions from analysis of PCSSR Framework (includes literature review and case study)	Author's recommendations to improve post-conflict self-recovery programming	Stakeholders concerned
1 - Economic	a - Budget per House	1a1 - Budget per House	Funding per shelter is based on donor constraints to maximize beneficiaries and not based on needs.	Donor funding should be flexible to allow the aid organization to adapt cost per household based on the needs.	Donors, Shelter Cluster
	b - Donors	1b1 - Donor Support	Donors vary considerably in terms of their acceptance of cash and self-recovery support modalities. Donors were consistently the largest barrier to further self-recovery programming.	Advocacy and donor engagement is recommended for agencies to highlight the benefits and risks of this modality in all contexts, but most importantly in post-conflict.	All
		1b2 - Time and Risk Concerns	Donors were hesitant to provide funding due to concerns over the timeframe and risk of the situation changing.	Programs should be designed to be flexible and adaptable to changing circumstances. Risk management is key in program planning and implementation.	Donors, NNGOs, INGOs
		1b3 - Demographic Changes Concerns	Donors were hesitant to fund projects based on concerns over contributing to demographic changes within the country.	Donors and the academic community should conduct further research to demonstrate any connection between demographic changes and self-recovery support programming.	Donors
		1b4 - Donor Priorities	Donors were more interested in projects with higher beneficiary reach than in long-term outcomes such as the social wellbeing of the population.	Donors and the academic community should better quantify the social benefits of self-recovery support programming. Aid organizations should host donors for site visits to demonstrate the benefits first-hand.	Donors, NNGOs, INGOs
		1b5 - Donor Trust	A lack of trust in local NGOs by donors prohibited some funding for self-recovery support projects.	Donors should embrace localization and shift their perspective on local NGOs. Many local NGOs are formed quickly after conflicts and will require time to meet the same standards and compliance requirements as large organizations. Local NGOs are often the best-positioned implementing partners for self-recovery projects since they have knowledge of the local culture and construction methods and are dedicated to the long-term vision.	Donors
		1b6 - Government Interaction	Donors are hesitant to fund programs which require interaction with the local government.	Donors should seek to build capacities of the local government alongside self-recovery support interventions. Specific risk identification and mitigation in certain locations is recommended instead of blanket policies.	Donors
		1b7 - Emergency Phase Constraints	Donors are hesitant to support longer-term shelter interventions due to the risk of re-emergence of the conflict.	The risk of re-emergence of the conflict should be evaluated against the potential impact of the work, considering the length of displacement and needs of the displaced population.	Donors
	c - Payments to Homeowners	1c1 - Payments to Homeowners	A best practice identified in Syria is to split payments to homeowners throughout phases of the project.	Iterative payments based on project milestones is a best practice that should be followed in post-conflict situations as it helps to avoid fraudulent beneficiaries and to monitor the project progress.	NNGOs, INGOs, IGOs
	d - Market	1d1 - Market	Price fluctuations are common in post-conflict situations and are most severe in the initial phases of the conflict.	Self-recovery programs should be adaptable and flexible to account for changes in the prices of materials and services throughout the execution of a project.	All
	e - Labour Market	1e1 - Labour Market	Post-conflict economies result in skilled labour leaving the country.	Technical capacity building should be included in programming to help restore skills to local labourers.	INGOs, IGOs
	f - Institutions	1f1 - Banking System	Banks are difficult to access and services are expensive, specifically for money transfers.	When relying on cash transfers, the state of the banks should be considered and factored into planning and budgeting.	All
2 - Social	a - Community Participation	2a1 - Community Participation	Community-level milestones associated with payments to homeowners can motivate the community to work together and rebuild social bonds.	Community-level targets for self-recovery projects is best practice that can be adopted in other contexts, depending on the conflicts/tensions in the community.	NNGOs, INGOs
	b - Vulnerable People and Women	2b1 - Prioritization	Vulnerable people and certain groups of women (such as single-headed households) often require the most support with post-conflict self-recovery.	Prioritization of women and vulnerable people is a best practice which should be continued in other post-conflict contexts.	NNGOs, INGOs
		2b2 - Additional Assistance	Additional assistance to vulnerable populations is beneficial including more frequent monitoring visits and assistance with finding contractors and labourers.	Additional technical support should be provided to vulnerable households. Contractor-led modalities should be considered for use in these cases since homeowners may not be able to complete repairs themselves.	NNGOs, INGOs
	c – Permanence and Stability	2c1 - Desire of Homeowners	Homeowners may not want to return home due to changes brought on by the conflict or due to psychological trauma associated with that place.	Self-recovery modalities should be accompanied by other shelter modalities as they will not be appropriate in all cases.	Donors, NNGOs, INGOs, IGOs
		2c2 - Further Research	There is a lack of research into self-recovery programming at large, specifically in terms of the social benefits which are harder to quantify.	Further research should be completed by aid organizations, academics, donors, and all stakeholders involved to facilitate and promote the benefits of this approach.	All
		2c3 - Young Men Engagement	Employing young men returning from the conflict in self-recovery works can facilitate reintegration in the community.	Where applicable, programming should seek to include vocational training and employment directed at young men.	NNGOs, INGOs
	d - Equity of Aid	2d1 - Beneficiary Screening	Households without the proper HLP documentation or at the proper damage level of their home can be left without aid.	Programming should provide alternate shelter options for those not eligible for self-recovery support.	NNGOs, INGOs, IGOs Shelter Cluster
		2d2 - Other Needs of Homeowners	Projects may not be initiated due to the perception that homeowners would choose to spend money on other things prior to shelter.	Multisectoral approaches should be implemented to ensure other needs of homeowners will be met as well. Further research should be done regarding how cash is spent in post-conflict situations.	All
3 - Governance	a - Local Authorities	3a1 – Varying Governing Bodies	Conflicts can result in various geographic areas being under different forms of control such as opposition and government-controlled areas.	Programs may require different designs in various areas of governance. Cooperation with the governing bodies in power is necessary in all cases to implement self-recovery support projects.	All
		3a2 - Local Authorities	Cooperation with local authorities is key in implementing self-recovery programs. Agreements may be written or verbal.	Supporting organizations should actively engage with local authorities throughout the project cycle and remain flexible in terms of the method of cooperation and agreement.	All
		3a3 – Government Permits	Cash-to-homeowner type projects were not approved by the Syrian government. Contractor-led projects took up to 6 months for approvals.	IGOs should advocate and build capacity with governments and authorities to allow permits for self-recovery project and to streamline approvals processes.	IGOs
	b - State Policies	3b1 - Bureaucracy	Non-government-controlled areas were easier to implement projects in due to a lack of normative/institutional barriers of government policy.	Due to the lack of oversight, in non-government-controlled areas, aid organizations assume more responsibility to build safely, verify HLP, and select beneficiaries appropriately.	All
		3b2 – Government Acceptance of Aid	Approximately three times more NGOs were operating in the opposition-controlled areas than government-controlled areas. This could be due to additional restrictions for aid work in government-controlled areas.	In other similar post-conflict contexts where aid must be delivered in government-controlled areas with many restrictions, government capacity building and advocacy should be prioritized through collaborative efforts.	All
	c - Shelter Sector Perception of self-recovery	3c1 – Operational Definition of Self-Recovery	Self-recovery is not a well-defined concept operationally and many organizations had varying understandings of it.	International guidelines should better define self-recovery and it should be done so in terms of levels since one definition cannot capture the entire scope of projects.	All
		3c2 – Relevance of Self-Recovery	In post-conflict situations, there are higher levels of women-headed households sometimes without construction skills. In most cases, even when given cash directly, women will hire contractors to complete the work.	Due to the lack of men, post-conflict self-recovery requires revision of modalities considering whether the homeowners themselves will complete the works. Lists of skilled labourers and guidance on hiring contractors should be provided.	All
	d - Capacity of Supporting Organization	3d1 - Organizational Knowledge	Previous organizational knowledge of self-recovery can be a key advantage, but local knowledge of the context is also important.	INGOs should capacity build with NNGOs, sharing previous relevant experience. In return, NNGOs should share their knowledge of local construction techniques.	INGOs, NNGOs
		3d2 - Managing Dispersed Projects	Aid organizations are challenged with the dispersed nature of self-recovery support, which differs from traditional support to sites or camps.	Implementing organizations should have robust logistical capabilities and staffing to monitor projects.	All
		3d3 - Competing Priorities	Dynamic post-conflict situations result in ad hoc emergency response being prioritized over self-recovery modalities.	Self-recovery should be considered within both emergency and recovery responses and should be designed to be flexible to changing situations in the context.	All
		3d4 - Control and Coordination	Contractor-led projects improved the control and coordination of these projects, especially for organizations working in cross-border aid.	Contractor-led projects are recommended when control and coordination are more difficult, especially when managing projects remotely from across a border.	All
		3d5 - Organizational Policies	Organizational policies such as Build Back Better may not be applicable in post-conflict responses due to a non-recurring damage modality.	Build Back Better policies should not be needlessly implemented when not required as this will only increase costs. Risks should be contextualized to the location.	All
		3d6 - Selection of Self-Recovery Modality	Contractor-led projects are more expensive but easier to control and more efficient for smaller standardized repairs such as doors and windows. Beneficiary-led repairs are cheaper, lead to higher levels of beneficiary satisfaction, but are more difficult to manage and control.	Both contractor-led and homeowner-led modalities should be used in conjunction, depending on the situation. Beneficiaries should have decision making power over the selected modality. Wherever possible, cash-to-homeowner projects should be chosen as these lead to the highest levels of beneficiary satisfaction.	NNGOs, INGOs
	e - Aid vs Development	3e1 - Aid vs Development	Self-recovery is often seen as a humanitarian action but has many ties to urban development. There lacks coordination between humanitarian and development sectors.	Coordination between the aid and development sectors should be prioritized.	NNGOs, INGOs, IGOs
	f - Capacity of Government	3f1 - Capacity of Government	The capacity of the government is greatly reduced throughout conflicts and its ability to control state-led reconstruction is diminished.	Capacity building of the government should be prioritized. Cross-imbedding government and aid staff in each others’ organizations can facilitate cooperation.	NNGOs, INGOs, IGOs
	g - International Coordination	3g1 - Intersectoral Approaches	Projects require integration with water, sanitation, and hygiene, health, transportation, and other sectors. There is a need for low-level intersectoral coordination bodies to accomplish this.	Intersectoral coordination mechanisms should be in place at various levels. International operational networks could be established to support low-level technical coordination.	NNGOs, INGOs, IGOs, Shelter Cluster
		3g2 - Shelter Cluster Guidelines	Shelter Cluster guidelines for rehabilitation and HLP greatly facilitate the coordination of self-recovery responses.	Shelter Cluster guidelines such as the rehabilitation and HLP guidelines should be replicated across other post-conflict contexts.	NNGOs, INGOs, IGOs, Shelter Cluster
		3g3 - Additional Guidelines	A lack of international guidance made projects more difficult to implement and convince donors of.	International guidelines for self-recovery support projects should be created. Additionally, guidance should be created on area-based approaches, urban vs rural self-recovery, conflict vs disaster self-recovery, repairing war-damaged buildings, and long-term shelter cash modality strategies.	Universities, IGOs
4 - Legal	4a - Tenure / HLP	4a1 - Lost Documentation	Homeowners lost tenure documents due to the conflict, thus making HLP verification difficult. The Shelter Cluster HLP Due Diligence Guidelines allowed for alternative methods of verification through utility bills or community leaders.	Cluster-level HLP guidelines should be replicated in other post-conflict responses including details for alternative documents that can be accepted for HLP. A complaint and review mechanism should be in place so homeowners can appeal decisions about tenure status.	All
		4a2 – Land Registries	Land registries can be targeted and destroyed during conflicts, compounding existing administrative issues before the conflict. This can result in long delays in tenure verification by the government. Support from NNGOs to help rebuild registries was beneficial.	Capacity building can be done to assist governments in rebuilding registries. Alternative forms of tenure verification can be used to rebuild these registries more expediently. Advocacy with governments to accept alternative tenure documentation temporarily could help facilitate project approvals.	All
	4b - Leasing Contracts	4b1 - Leasing Contracts	Exchanging housing repairs for free rent for an IDP family for a certain period of time is an approach that has been effective.	Repairs-for-rent modalities should be replicated in other post-conflict responses.	Donors, NNGOs, INGOs
5 - Contextual	5a - Conflict and Security Situation	5a1 – Prolonged Conflicts	Repair and rehabilitation projects (including self-recovery modalities) can become more relevant later in a conflict as other shelter options have been exhausted, housing stocks have been reduced, and people have started to return home.	Self-recovery programming may be most relevant to implement at later stages of a prolonged conflict, but it greatly depends on the context.	All
		5a2 – Changing Conflict Situation	Projects can take up to one year to complete. With the changing conflict situation, this creates a high risk of projects not finishing on time.	Projects should be designed with flexibility to adapt to changing conflict situations and risk assessments should be a part of project planning	All
	5b - Returnees	5b1 - Reasons for Return	Returnees may return due to the conflict situation becoming more stable, the lack of sufficient aid provided in overcrowded camps, and, in the case of Syria, the COVID-19 crisis in the camps.	Supporting organizations should monitor returnee inflows and plan self-recovery programs strategically.	All
	5c – Access of Aid Organizations	5c1 – Organizational Security Policies	Aid organizations have different security policies. UN Agencies are not able to access Syria from Turkey due to security policies, whereas many of the other INGOs and NNGOs are able to.	Smaller organizations with more flexible security restrictions are better suited to conduct self-recovery support in post-conflict settings. Organizations with access restrictions can consider using implementing partners.	All
		5c2 - Cross-border Aid	Security restrictions lead to organizations conducting cross-border support in a neighbouring country, which causes monitoring and controlling issues. Cell phone application technology to communicate with implementing partners facilitated monitoring works.	Innovative methods to monitor and control projects remotely will be beneficial to cross-border management of self-recovery support, depending on internet connectivity. Information, education, and communication (IEC) materials could be used to support homeowners with construction remotely.	All
6 - Technical	6a - Building Codes	6a1 - Building Codes	Due to the absence of building codes in some post-conflict responses, aid organizations follow their own standards.	The Shelter Cluster should standardize building codes for organizations to follow to ensure a standardized response. Where possible, local building codes should be followed and improved as required such as through capacity building with private sector partnerships.	NNGOs, IGOs, Shelter Cluster
	6b - Type of Damage	6b1 - Damage Classification	Various organizations have differing scales for damage classification.	International guidelines should standardize damage scales for post-conflict situations for ease of use and information sharing.	All
		6b2 - Lack of Expertise in Conflict-Damage	There is a lack of understanding, technical expertise, and technical guides and manuals for repairing conflict-damaged buildings.	Research should be conducted into how ordnance affects structures and how to repair these structures. Technical manuals should be developed for aid organizations to support programming.	All
		6b3 - Looting	Looting was noted as a major cause of secondary damage to homes.	Self-recovery programs should focus on houses that have not been left unattended for long periods of time.	NNGOs, INGOs
		6b4 - Structural Damage	Structural repairs are not permitted by aid organizations due to the safety issues with these repairs and the perception that this is reconstruction, which should be done by the government. This can result in the most-affected households not receiving support.	Local NGOs should be permitted to conduct structural repairs if engineers are overseeing the works. For international organizations, further advocacy could be conducted to allow for some structural repairs to be within scope. Private sector partnerships could help to fill gaps in engineering resources for aid organizations.	Donors, IGOs, Shelter Cluster
	6c - Infrastructure Systems	6c1 - Infrastructure Systems	Housing repairs were not possible in some cases due to the lack of infrastructure systems such as roads, water systems, electricity, and sewage is integral to self-recovery projects.	Infrastructure repairs should be done in conjunction with housing. One method to accomplish this is through area-based approaches, or the Settlements Approach, where one organization takes responsibility to ensure a multi-sector response.	All
	6d – Supporting Organization's Technical Competency	6d1 - Professionals on Staff	Engineers and architects on staff greatly facilitate the technical aspects of self-recovery support projects.	Organizations should choose the level of self-recovery support (doors and windows vs. walls and roofs) depending on their technical capacities. Contracting this support from local professionals is possible.	All
		6d2 - Quality Control	Due to security restrictions, there may be difficulties in controlling quality of works.	Quality control mechanisms such as thorough contracts, project completion inspections, and third-party quality audits can be used.	All
	6e - Other Shelter Response Modalities	6e1 - Housing Stock	The repair and rehabilitation modality is limited by the number of damaged houses, their damage level, and location. Unfinished housing from before the conflict is sometimes used as another source for housing stock.	Self-recovery support strategies should be based on all available options including the repair of existing homes, completion of unfinished housing from before the conflict, and other new construction options such as transitional shelters.	Donors, NNGOs, INGOs, IGOs, Shelter Cluster
	6f – IEC Materials	6f1 - IEC Materials	IEC materials such as easy instructions on how to repair certain damages or instructions on which materials to buy have been successful.	IEC materials should be shared amongst NGOs and resources like the Global Shelter Cluster’s IEC Compendium should be expanded with post-conflict content. IECs are especially useful to organizations in cases of cross-border support.	All
	6g - Building Safety	6g1 - UXOs	There is a risk of houses containing unexploded ordnance (UXO)s which presents a significant safety risk for all involved.	UXOs require specialist support to remove. NGOs should collaborate with local authorities to facilitate this.	NNGOs, INGOs

## Discussion: key areas for action

By analyzing the Recommendations table, five Key Areas for Action were identified which represent cross-cutting themes of recommendations to better support self-recovery in post-conflict situations. These Key Areas for Action are maximizing implementing organizations’ capacities, contextualizing risks, increasing adaptable and flexible programming, addressing the social dimension, and improving international coordination.

### Maximizing implementing organizations’ capacities

The first Key Area for Action is to maximize implementing organizations’ capacities. Because post-conflict situations often present significantly reduced local government capacities, implementing organizations accept increased responsibilities that, in natural disaster emergencies, often the government would assume. This includes confirming HLP documentation of homeowners, ensuring adherence to building standards, and properly screening beneficiaries (Ohiorhenuan 2011, p. 9; Davis 2015). This necessitates a strong and capable implementing organization and means that opportunities should be taken to maximize the capacities of implementing organizations involved in this work. Two factors were identified to maximize implementing organizations’ capacities: knowledge sharing between local and international organizations and increasing efficiencies in monitoring and controlling.

To understand the benefit of knowledge sharing between local and international organizations, it is first necessary to understand what organizational knowledge is possessed by these organizations. In terms of larger INGOs, organizational knowledge is largely in the form of previous organizational experience in self-recovery support projects, largely from disaster contexts. In fact, this previous experience was noted as a significant facilitator of self-recovery support projects being initiated in Syria. Without this previous experience, they were less likely to attempt this modality for the first time in post-conflict situations due to the complexities involved^2^. NNGOs, however, often do not possess experience in previous self-recovery programs. In fact, many NNGOs are often founded shortly after a conflict begins and, thus, do not have any previous organizational knowledge whatsoever. What NNGOs possess, however, is a wealth of experience in the local construction sector and vernacular architecture since many of their staff are local built environment professionals.

Understanding the differences in organizational knowledge between NNGOs and INGOs reveals opportunities for knowledge sharing. Knowledge sharing can help NNGOs and INGOs to fill each other’s knowledge gaps, and thus, maximize their capacity to support self-recovery. One best practice for knowledge sharing that was identified in Syria is for NNGOs to train INGOs in local construction methods and vernacular architecture. INGOs also have knowledge to share such as lessons learned from previous self-recovery support projects. Although this was not noted to be happening in Syria, this should be done to support NNGOs that have little organizational experience (Table 3 rec 3d1). Additionally, INGOs should consider making their organizational knowledge more accessible to other NGOs. This could be done by creating self-recovery project databases coordinated through an international body such as the Global Shelter Cluster (Table 3 rec 6f1). These databases can contain guidelines, data, and lessons learned, much of which already exists at the INGO level but is not accessible by smaller NGOs. This knowledge sharing would help to maximize implementing organizations’ capacities to fill gaps in knowledge on the sides of both local and international organizations.

Finding efficiencies in the monitoring and controlling process can also maximize implementing organizations’ capacities. Monitoring and controlling was one of the main barriers for NGOs supporting self-recovery since beneficiaries are often dispersed and in areas that are difficult to access by implementing organizations. Because of this barrier, many large INGOs disregard self-recovery projects and opt for more traditional shelter support modalities that are easier to control. This can include IDP camps and transitional housing settlements. Finding efficiencies in monitoring and controlling would, thus, enable organizations to conduct self-recovery support more easily.

The main barriers identified regarding monitoring and controlling were coordination with local partners, communication with homeowners, controlling quality, and having the resources to conduct required visits and inspections. One strategy identified to address these difficulties was implementing quality control mechanisms. This can include thorough contracting procedures with contractors, project completion signoffs with all stakeholders, third-party quality audits, and innovative mobile phone applications which allow for remote project monitoring (Table 3 rec 3d2, 3d4). The use of local implementing partners was effective as well, especially for INGOs which could not access the project locations due to security policy restrictions (Table 3 rec 5c2). The benefits of using local implementing partners include the experience of these partners in local construction methods, the removal of some coordination work from the INGO, and the positive contribution to the independence of local NGOs who will maintain a lasting presence into the future. Some organizations though have strict ethical policies against the use of local implementing partners since they perceive this as putting their local partners at more security risk than they are willing to assume themselves.

Another opportunity for increasing efficiency in monitoring and controlling is through the proper selection of the self-recovery response modality. Various response modalities can be easier to monitor and control depending on the circumstances, and if NGOs can select responses accordingly, they will act more efficiently. Of the participants in this research, only 20% of organizations conducted cash-to-homeowner projects with 80% choosing contractor-led projects for the main reason of these projects being easier to monitor and control. As one IGO explained, “it is easier to chase one contractor than to chase 1000 landlords” (anonymous participant, Skype interview, May 05, 2020). It is worth remembering from literature, however, that in Bosnia, contractors were chosen for the similar purposes of speed and project control, but these benefits were never actually seen compared to the homeowner-led projects (Barakat 2005, p. 165). This makes it clear that selecting the proper response modality requires an analysis of the specific circumstances. It is observed that contractor-led projects are easier to monitor and control when NGO access is limited, such as in cross-border aid, whereas cash-to-homeowner projects are easier when the NGO can regularly be on site and monitor and control directly. Since most participant organizations were working with significant access restrictions, it seems appropriate that 80% of them were choosing contractor-led projects in this case. For other conflicts, the context would need to be considered, especially considering access restrictions, to select the appropriate modality. Additionally, contractor-led projects were noted by participants to be easier to control for small, standardized repairs such as doors and windows whereas cash-to-homeowner were optimal for non-standardized repairs. It is recommended that organizations select their appropriate response based on these recommendations and, in this way, their capacities will be maximized to provide the most support possible (Table 3 rec 3d4, 3d6).

### Contextualizing risks

The second Key Area for Action is to ensure the proper contextualizing of risks by both donors and NGOs. Contextualizing risk means that risks should be assessed based on the actual context and should not be assumed based on other experience. This must be done at multiple levels. For instance, post-conflict contexts must be assessed without preconceived notions from natural disasters. Similarly, the Syrian context must be assessed without preconceived notions from neighboring countries or other conflicts. Additionally, it means assessing risk iteratively based on a situation that can change considerably over time. Contextualizing risks allows a better-informed assessment of actual risks to avoid the common tendency of inclining towards being more risk averse and, thus, needlessly excluding many people in need. Risks must be contextualized within four areas: HLP documentation, structural repairs, demographic changes, and natural disasters.

HLP documentation is one area where donors and implementing organizations must adequately assess risk and trade-offs. As has been shown previously, HLP documentation is often hard to confirm in post-conflict situations. Despite this, most NGOs have clear policies against self-recovery support if tenure cannot be confirmed (Seneviratne et al. 2013; Davis 2015). In Syria, this has resulted in significant amounts of people being excluded from support. Recently, somewhat more flexible guidelines have been implemented to address this such as the Global Shelter Cluster Turkey hub’s HLP Due Diligence Guidelines and, in the government-controlled areas, the recent acceptance of alternative documentation for HLP. These flexible HLP guidelines are crucial facilitators in post-conflict situations to ensure more beneficiaries can be reached. Many organizations, however, still say these do not go far enough and continue to be too exclusionary. As a member of the UNHCR Strategic Advisory Group said: “many people are living in reception centers, unfinished buildings, and damaged buildings, but organizations cannot do anything for them because of HLP rights” (A. Dehny, skype interview, April 16, 2020). As a conflict progresses, risks must be iteratively assessed and, when there is such a substantive demand for shelter, as in Syria, trade-offs must be reassessed (Table 3 rec 4a1).

The risk of conducting structural repairs is another area which requires adequate contextualization to the post-conflict environment. Structural repairs come at a heightened risk because they involve repairing structural components such as load-bearing walls, columns, and slabs, which, if not done properly, will cause building collapse. In some ways, the case study showed that this risk is being properly contextualized in Syria as the current prohibition on structural repairs is partially based on the lack of understanding of how buildings are damaged in conflicts. In comparison, in natural disaster contexts, structural repairs are possible because there is an understanding of the effects of earthquakes on buildings. In this way, risk is being contextualized to the post-conflict environment and it has been decided that no risk will be taken regarding structural repairs. Despite this reality, it must be questioned as to why this is being accepted and more is not being done to reduce risks to make structural repairs possible. Many organizations lamented the fact that they could not conduct structural repairs with one INGO member stating, “the ones who need most help, we can’t help them, so we focus on the ones who need less help” (INGO, skype interview, March 31, 2020). What is required in this case, is more research about how conflict ordnance damages buildings. This knowledge will allow for proper risk assessments to be done in the field, which will help to identify more easily those with non-structural damage and those with repairable structural damage. Further integration of humanitarian aid with local professional engineering agencies and private-sector partnerships are a necessary step to implementing structural repairs (Table 3 rec 6b2, 6b4).

There is another risk relating to structural repair, however, that is not being properly contextualized; the risk of this work being perceived as reconstruction, i.e., permanent. In some cases, participants noted that any structural work was not permitted, even if they had the professional expertise to do so, due to this perception. Donors specifically were noted to be very risk averse regarding structural repairs since reconstruction work is meant to be done by the government. If an aid organization were found to be doing this, it could receive backlash from the government, and this could potentially impact its ability to continue to operate in the area. This risk is not properly being contextualized, though, since it is being left to each individual organization to consider, and it is being interpreted very differently. For instance, some organizations noted that they chose not to build metal roofs due to this being perceived as structural repairs. Other organizations determined that it was fine to construct concrete pads and brick walls which are much more permanent interventions. The result of this subjective interpretation is that beneficiaries are often provided less adequate shelter, not because of funding or technical issues, but simply because of the organization’s designation of what structural repairs consists of. This paper does not argue that reconstruction should be allowed as part of an aid response, but simply that standardization be achieved between aid organizations. Guidelines could be produced to define what the line between reconstruction and repair is, so that it is clear. These guidelines could be developed by the Global Shelter Cluster, in coordination with local authorities. Finally, it is also recommended that, if possible, in cases of prolonged conflicts where there is such an immense demand for adequate shelter, that risk be iteratively assessed to account for changing realities on the ground and loosened accordingly to allow for some basic structural work to be done (Table 3 rec 6b4).

Another risk that must be contextualized is the risk of shelter interventions causing demographic changes. Many organizations noted that donors restrict self-recovery support due to the fear of being accused of contributing to demographic changes within the country. This risk, although warranted, must be contextualized to the reality on the ground. Participants argued that the true reason for demographic changes in Syria is the conflict itself, and not the aid. Additionally, donors may be misinformed about the self-recovery process since self-recovery support is mostly directed at homeowners who have lived in their homes since prior to the conflict, meaning no demographic changes would be created. While self-recovery programs do also support IDPs in the cases where homeowners rent out repaired homes to IDPs, it is unlikely that these IDPs would settle permanently in a new area simply because of the self-recovery support; the reality is much more complex. In prolonged conflicts such as in Syria, where some IDPs have been displaced for over a decade, donors must continually reassess the situation and perhaps loosen their risk policies regarding demographic changes (Table 3 rec 1b3).

The risk of natural disasters must also be contextualized in the post-conflict context as it has a significant impact on the design of shelter responses. It must be understood that in post-conflict situations, there is sometimes no requirement for changes in building techniques. This is because the existing house might have been designed perfectly in accordance with the natural disaster risk in the area but was destroyed due to military ordnance; a non-recurring threat. It was noted by participants that some organizations’ leadership do not adequately contextualize this risk and implement unnecessary requirements related to building back better which have been developed from previous organizational experience. By doing so, funds would not be spent in the most efficient way to reach the most beneficiaries. Thus, it is important that organizations and donors contextualize all risks that might not be applicable in post-conflict situations to ensure they are not coming with, what Barakat (2005) describes, “preconceived practices and assumptions… which override local conventions and capacities” (p.159) (Table 3 rec 3d5).

### Increasing adaptable and flexible programming

The third Key Area for Action is to increase adaptability and flexibility of post-conflict self-recovery support. A lack of flexibility is a common failure of post-conflict shelter interventions. As Barakat (2005) explains, “external interventions often lack the necessary practical adaptability and flexibility to deal with the dynamics and high levels of uncertainty found in post-conflict environments” (p. 159). Flexibility and adaptability should be implemented within all aspects of programming, but specifically within three domains: funding, scheduling, and scope.

Funding requires flexibility and adaptability at multiple levels including the donor level and the implementing organization level. At the donor level, it was noted that the funding for self-recovery projects is often provided with a rigid, prescribed, per-shelter amount and that this amount is insufficient for most repairs. This strategy is likely employed so that donors can control and maximize the number of beneficiaries they are reaching. The consequence of this, however, is that implementing organizations must significantly limit their support to align with the prescribed funding amount, which usually equates to only minor repairs. Although maximizing the number of beneficiaries is good in some cases, it is the implementing organization, not the donor, in the best position to make this determination. Implementing organizations on the ground should have the power to determine how funding is distributed for the maximum benefit. Donors should place less importance on quantifying beneficiaries and consider increasing the flexibility of their funding to allow the implementing NGOs the freedom of determining how that funding is distributed (Table 3 rec 1a1). Participants also noted that funding is sometimes restricted to material or technical assistance and that cash-based assistance is not permitted, thus significantly limiting support options. Donors should consider more flexibility in terms of their funding to allow cash-based support for self-recovery which will facilitate implementing organizations in variable responses (Table 3 rec 1b1).

Adaptability of funding is also required within implementing organizations’ programming. Participants noted that their organizations’ internal processes did not allow for funding and budgets to be adaptable throughout the project. In post-conflict situations, the market prices for materials can fluctuate greatly on a weekly basis and this means that bills of quantities and budgets will need to be adapted throughout the project. Many organizations do not account for this, which results in projects being either over budget or left unfinished. Self-recovery project budgets must be adaptable and reviewed regularly (Table 3 rec 1d1).

Scheduling is another area requiring flexibility and adaptability. Self-recovery projects may have long timeframes, up to 1 year from the initial selection of beneficiaries to the final completion of construction. In post-conflict situations, this leads to projects often being disrupted by changes in the conflict throughout the construction. Participants noted that, due to this risk, donors were hesitant to initiate self-recovery projects. If adaptability and flexibility were included into scheduling, however, self-recovery projects could be modified, paused, or rescheduled depending on changes in the conflict. This could increase the success of self-recovery approaches despite the challenging situation and potentially help to convince donors to initiate these projects (Table 3 rec 1b2).

Project scope is another area in which implementing organizations and donors must remain adaptable and flexible. Post-conflict situations evolve rapidly and there is often a fluidity between the emergency and recovery phases. This necessitates consideration of a wide scope of shelter response modalities and often the combination of multiple modalities. These may include tented camps, transitional shelters, collective centers, cash-for-rent programs, and self-recovery support. Within each selected modality itself must also be an element of flexibility so that the modality can evolve over time with changing circumstances. Participants noted that this was not often the case, as sudden changes in the situation, such as events requiring rapid emergency responses, frequently resulted in self-recovery projects simply being canceled. This shows a lack of flexibility within the program design. Although emergency response rightly takes priority in these circumstances, self-recovery programs must be designed to be adaptable to ensure they can continue progressing throughout an evolving conflict situation (Table 3 rec 1b1, 3d6).

### Addressing the social dimension

The fourth Key Area for Action is to address the social dimension of self-recovery programs. Although self-recovery approaches have produced superior social outcomes than traditional shelter response modalities, there are still issues relating to the understanding of the social dimension in post-conflict situations. The social dimension must be addressed in three areas: intended beneficiaries, program goals, and further research.

Firstly, there is a current misunderstanding regarding the social characteristics of the intended beneficiaries of self-recovery programs in the post-conflict context. The literature that self-recovery has been based on is from the disaster context and the idea that homeowners have the skills and ability to rebuild their homes (Davis 1978). Although, to some degree, this remains true in post-conflict situations, it does not appear to be as relevant as in natural disasters. This is because in post-conflict situations, many of the men are fighting, have fled, or have been killed, and men are often traditionally the ones to do construction work (Corsellis and Vitale 2005, p. 50). This is the case in Syria, as the number of women-headed households is high. This means that many of the beneficiaries of self-recovery programs in Syria are women, who often do not have the construction skills to complete repairs themselves (UNHCR 2014). This must be considered when implementing self-recovery programs in post-conflict situations as these women-headed households will usually not conduct the labor themselves. In fact, participants noted that almost all women-headed households who were given cash decided to contract the work to local laborers.

Additionally, self-recovery programs in Syria are usually targeted towards vulnerable people which might be women with children, the elderly, or the disabled. These vulnerable people are also not likely to be able to complete construction work themselves. This is an important social consideration specific to post-conflict situations which goes somewhat against the initial concept of self-recovery as applicable in disaster contexts. The shelter sector must reconsider its basic understanding of intended beneficiaries for self-recovery support in post-conflict situations and must build this into program planning and design (Table 3 rec 3c2).

The social dimension must also be included within the determination of self-recovery program goals. As Barakat (2005) criticizes from other past conflicts, post-conflict housing programs tend to be project-driven, short-term focused, and output-driven rather than outcome-driven (p. 158). Post-conflict housing project goals are often overly focused on indicators, quantifiable metrics, and statistics rather than more social-oriented goals such as privacy, health, stability, livelihoods, and security. Barakat notes the requirement for not only physical shelter interventions, but also for social ones, such as capacity building (pp. 158-164). The Syrian case study shows that Barakat’s criticisms are still valid. Participants noted donors as being too output-focused, short-term in thinking, and placing too much importance on numbers of beneficiaries. One INGO participant indicated that sometimes to appease donors, implementing organizations resorted to first conducting a superficial project that could increase their beneficiary count, before they could actually focus on completing the work they believed would have the greatest impact to long-term outcomes. Additionally, participants noted the specific lack of capacity building initiatives paired with self-recovery programs, especially regarding the training of laborers. There is a clear need for the social dimension to be included within self-recovery project goals to ensure they are outcome-driven and long-term oriented and include important capacity building initiatives (Table 3 rec 1b4).

The social dimension must also be included in further research regarding self-recovery support. Participants noted the absence of research regarding the social benefits of self-recovery interventions and the difficulties in proving the benefits of these projects to donors without this research. Some aid organizations conduct their own internal research and data collection regarding social benefits; however, this information does not seem to be shared widely within the sector. Additionally, it was noted by participants that social benefits are hard to quantify, which makes internal research difficult. Further research regarding methods of quantifying and identifying the social benefits of self-recovery projects would be beneficial helping to educate all stakeholders, including donors (Table 3 rec 2C2).

### Improving international coordination

The fifth Key Area for Action is to increase international coordination to support self-recovery in post-conflict situations. This means improving coordination at all levels and among all stakeholders including universities, donors, NGOs, IGOs, the Global Shelter Cluster, and governments. Several areas in which enhanced international coordination can improve self-recovery support are observed: standardization of self-recovery terminology and guidelines, donor engagement, international operational networks, and private sector partnerships.

International coordination could assist in the standardization of self-recovery terminology and processes, which is currently lacking. Many participants were not familiar with the concept of self-recovery until it was explained and they were able to understand based on their own organizational terminology. The term most used describing self-recovery in Syria was observed to be *self-help* since this is how it is phrased in the Global Shelter Cluster guidelines. Although terminology is somewhat irrelevant in terms of operations, it does become relevant when information sharing is considered. It was noted that many organizations have internal self-recovery guidelines, standards, and case studies, but that these are not shared. With a standardized terminology in place, these could be more easily developed and shared (Table 3 rec 6f1, 2c2). Additionally, as has been shown in this study, there are varying shelter responses in post-conflict situations that can be accommodated within the current definition of self-recovery. A clearer definition is required to determine exactly which post-conflict shelter responses are considered as self-recovery support. Additionally, if it is accepted that self-recovery support must be defined in terms of levels, as classified in the case study of this paper, these levels must be defined and accepted by stakeholders (Table 3 rec 3c1). This requires international coordination between universities, NGOs, and IGOs.

Agreed-upon terminology and definitions will also facilitate the establishment of self-recovery guidelines. Although some self-recovery guidelines exist at the organization level and cluster level, there are none at the international/strategic level. Some organizations such as the Promoting Safer Building Working Group are working on such international guidelines. They acknowledge that currently “there are no guidelines, nor tools, nor even guiding principles to support the implementation of self-recovery projects” (Promoting Safer Building Working Group 2020). The scope of this working group is mainly in disasters, however, so further guidelines specific for post-conflict situations have yet to be addressed. Participants supported the idea of guidelines being created, with 75% responding favorably to the idea. Other guidelines were also requested by the majority of participants such as technical guidelines for repairing war-damaged buildings and guidelines on long-term cash modality strategies in post-conflict contexts (Table 3 rec 6b2). International coordination could facilitate these guidelines, to ensure standardization across universities, NGOs, and IGOs (Table 3 rec 3g3).

The next area for increased international coordination is in donor engagement regarding self-recovery projects. This should not be done with a view to promote self-recovery over other methods, but simply to help donors to understand the real benefits and limitations of this modality. NGO participants noted the requirement to constantly convince donors of the effectiveness and merits of self-recovery methods, due to the lack of knowledge of donors regarding this modality. This was noted to vary between specific donors. International shelter sector stakeholders could be beneficial in this process by informing the perception of donors at a high level. This could occur through increased research into these modalities, through advocacy and increased discussion at the international level, and through direct donor engagement and education (Table 3 rec 1b1, 2c). One participant noted that their donor was initially hesitant to fund self-recovery projects but that by bringing the donor to the site of one of these projects, the positive impact was immediately seen, and funding was granted. Further efforts such as this could be done at the international shelter sector level and would help with the donor community’s acceptance of these methods (Table 3 rec 1b4).

There is also an opportunity for the formation of international operational networks to facilitate self-recovery support operations. These could include academic institutions, private companies, aid and development organizations, intergovernmental organizations, and government agencies. Participants noted the need for such networks due to the intersectoral nature of self-recovery work and the lack of international coordination bodies at lower levels. Intersectoral approaches are required since repairing a house in a post-conflict situation requires significant lateral coordination for rubble removal, repairing roads, reconnecting water and sewage lines, reconnecting electricity, and potentially UXO disposal. International operational networks could be formed to as a coordination mechanism between all stakeholders involved in these activities. Currently, various organizations are accomplishing this on their own without any coordination bodies in place. This act of connecting all actors involved in post-conflict response could also begin rebuilding connections and systems that could become the foundation for building capacity back into local institutions (Table 3 rec 3b1). As Barakat (2005) noted, this is the real area that international coordination can support post-conflict situations, through the strengthening of institutions and systems (pp. 159-164).

International coordination in the establishment of private sector partnerships could also provide an opportunity to increase support for self-recovery in post-conflict situations. Specifically, international private engineering firms could partner with implementing organizations to support self-recovery with specialized engineering work. Some participants expressed that they would not be comfortable with their organization leading structural repair work. These participants believed that structural work should not be in the scope of aid organizations but should be completed by private engineering firms. It is possible that international private engineering firms could contribute great value to post-conflict situations, depending on the capacities of local engineers. The previously discussed barriers regarding structural repairs and the perceptions of who should lead reconstruction would have to be resolved before this could occur. If this issue can be addressed, though, international engineering firms such as Arup Group and Mott MacDonald could be good partners for NGOs since they already have international development departments and have some experience in humanitarian engineering (Table 3 rec 6a1, 6b4).

As part of this study, one interview was conducted with Arup Group and determined that although these types of partnerships could be possible, there are significant barriers from the perspective of engineering firms. These include security of their personnel, speed of response, financing, and normative constraints. One area identified which has immediate potential for collaboration is in the creation of IEC materials. These materials, which could take various forms such as pamphlets or manuals, could provide written and visual guidance to homeowners in conducting repairs themselves. Another potential opportunity identified was in online, or remote, engineering (Table 3 rec 5c2). Remote engineering is a burgeoning new field which consists of using technologies such as drones and cameras to allow engineers to assess structures remotely. This is becoming especially relevant today as COVID-19 has recently forced engineering firms to rethink how work can be done at a distance. Innovations in international coordination such as this could help to close some of the gaps and break down some of the barriers currently holding self-recovery back in post-conflict contexts.

## Conclusion

This research has attempted to bring some clarity to the complex process of supporting self-recovery in post-conflict situations and identify ways to improve this support. This was accomplished through the identification of common factors affecting the implementation of these projects and categorization of these factors into a framework. From this framework, recommendations and Key Areas for Action were identified. These recommendations and Key Areas for Action are hoped to assist implementing organizations, donors, and other stakeholders in their understanding of these projects and how best to enhance them.

The framework, recommendations, and Key Areas for Action within this study require further development from other contexts. Not all of the factors and recommendations will be applicable to all post-conflict situations as some may be unique to the Syrian context. Due to the complexities inherent in post-conflict situations and in self-recovery programming as a modality, further research from other contexts is required to prove the framework and recommendations. Additionally, the framework and recommendations should be expanded to include other stakeholders’ perspectives which were not able to be included here. This includes local authorities and governments as well as, the most important stakeholder involved, the homeowners themselves. Although the limitations of this study placed the focus on implementing organizations, there must be further work to understand homeowners’ perceptions about this type of support and to identify gaps in support from their perspective.

The case of Syria is important to continue studying. Self-recovery modalities are becoming increasingly utilized in Syria, being propelled by a parallel trend in the sector towards increased returnees and increased cash-based responses. Many organizations are currently struggling with these projects against the barriers identified in this study; however, some are creating new innovative approaches and best practices which should be considered for other post-conflict responses. Other post-conflict contexts must also continue to be studied to broaden the understanding of post-conflict self-recovery to new contexts.

Further research is also required most urgently to address the social and technical considerations of post-conflict self-recovery. Key social benefits of self-recovery support such as its impact to health, livelihoods, and social cohesion are important to highlight for the post-conflict context, where these benefits would have a great impact to post-conflict recovery. On the technical side, key barriers continue to prohibit this modality due to the lack of understanding of the damage modality of housing due to ordnance. Further understanding of this will support more comprehensive repairs to damaged buildings for greater long-term outcomes for the affected populations.

## Data Availability

Data sharing is not applicable to this article as no datasets were generated or analyzed during the current study.

## Footnotes

1 This research will use the term *post-conflict* to describe humanitarian responses following conflict situations, although it is acknowledged that often conflicts may still be ongoing and that the line between conflict and post-conflict can be ambiguous.

2 Previous experience in self-recovery support from natural disaster contexts must be evaluated as to its appropriateness in post-conflict contexts. It was noted by participants that some large organizations improperly applied experience from previous disaster contexts in Syria. This included policies regarding *Building Back Better* and *Disaster Risk Reduction*, which are concepts not necessarily applicable in the post-conflict environment.
